# Uterotropic response to the antidepressant fluoxetine (Prozac) is dependent on a functional serotonin transporter and circulating serotonin

**DOI:** 10.1038/s41598-025-27477-w

**Published:** 2025-12-10

**Authors:** Rafael R. Domingues, Jessica Fox, Emma Day, Milo C. Wiltbank, Laura L. Hernandez

**Affiliations:** 1 Department of Animal and Dairy Sciences, University of Wisconsin-Madison, Madison, WI USA; 2 Present Address: Department of Animal Sciences, The Ohio State University, Columbus, OH USA

**Keywords:** Endocrine disruption, Estrogen signaling, Selective serotonin reuptake inhibitor, Serotonin transporter, Uterotrophic assay, Reproductive biology, Molecular biology

## Abstract

Selective serotonin reuptake inhibitors (SSRI) are the most used antidepressants. However, they appear to pose a role as endocrine disrupting agents, particularly related to estrogen signaling. We sought to investigate the in vivo effects of fluoxetine, the most well-known SSRI, on uterine dynamic using uterotropic bioassays. In prepubertal mice, lack of increase in uterine weight in a three-day uterotrophic assay indicated fluoxetine does not directly bind estrogen receptor to elicit estrogenic effects. Conversely, in virgin, sexually mature mice and pseudopregnant mice, fluoxetine (2 mg/kg/d) elicited uterotropic response with increased uterine weight. Further, we observed increased uterine thickness, increased epithelial height, increased endometrial grands, and altered uterine gene expression (upregulated Alkp and downregulated Igfbp3) consistent with the uterotropic response and suggestive with modulation of estrogen signaling. In mice with genetic ablation of the serotonin transporter (Slc6a4^−/−^, target site for SSRI) and depleted peripheral (nonneuronal) synthesis of serotonin (Tph1^−/−^), fluoxetine did not elicited uterotropic response. Therefore, the uterotropic response to fluoxetine is dependent on inhibition of serotonin transporter function and mediated by circulating serotonin.

## Introduction

Selective serotonin reuptake inhibitors (SSRI) are the main class of antidepressants used to treat multiple psychological conditions including depression, anxiety, and obsessive-compulsive disorder^1–3^. The use of SSRI has increased over the last several decades with 13.2% of adults using antidepressants in the US. Noteworthy, the frequency of use of antidepressants is two-fold greater in women compared to men as 17.7% of adult women are prescribed antidepressants^4^. After the COVID-19 pandemic the use of antidepressants increased 11.7% in adolescents and 7.5% young adults^5^. The most consumed antidepressant in adolescents is the SSRI fluoxetine. Nevertheless, SSRI use is associated with reproductive side effects that include sexual dysfunction, amenorrhea, and galactorrhea, in addition to adverse pregnancy outcomes^6–10^. The incidence of sexual dysfunction in women taking SSRI is up to 80% and women have more severe SSRI-induced sexual disfunction compared to men^11^. Given that sexual dysfunction is often due to endocrine causes, including endocrine disruption^12^, understanding the effects of SSRI on endocrine homeostasis can provide insight into the role for SSRI on sexual dysfunction.

Inhibition of the serotonin transporter (SERT, encoded by Slc6a4 gene) by SSRI occurs in the brain and in the periphery resulting in increased serotonin signaling throughout the body^13–15^. At the tissue level, inhibition of SERT prevents serotonin transport into the cells for degradation prolonging serotonin’s presence in the extracellular space where it binds to its cell-surface receptors^1,16^. Additionally, SSRI increase platelet-free circulating serotonin concentrations^15^. Several studies have reported that SSRI modulate steroid synthesis and signaling in vivo and in vitro^17–23^. However, whether the effects of SSRI on steroidogenesis are mediated by a mechanism involving inhibition of SERT and modulation of serotonin signaling or through an off-target (off-SERT)^24^mechanism remains to be elucidated. Additionally, despite the potential of SSRI to act as endocrine disrupting agents and thier impacts on the regulation of reproductive cyclicity and behavior^25–27^, direct evidence for a role of SSRI modulating uterine dynamics in vivo is limited.

In a previous experiment in our lab (unpublished), we observed increased uterine weight in 75% (6/8) of nonpregnant female mice mated to a fertile male and treated with fluoxetine (2 mg/kg/d), whereas untreated, nonpregnant mice had normal uterine weight. Increased uterine weight is a common response to compounds with estrogenic actions. Indeed, uterotropic bioassays have been used for decades as a screening tool to assess biological activity of various compounds, including drugs and endocrine-disrupting chemincals, that may modulate estrogen signaling^28–30^. Therefore, we aimed to investigate the in vivo effects of fluoxetine treatment on estrogen signaling performing a series of uterotropic assays in mice to unravel the role of SERT and serotonin on the SSRI-induced endocrine disruption.

## Results

### Experiment 1

**Fig. 1:**
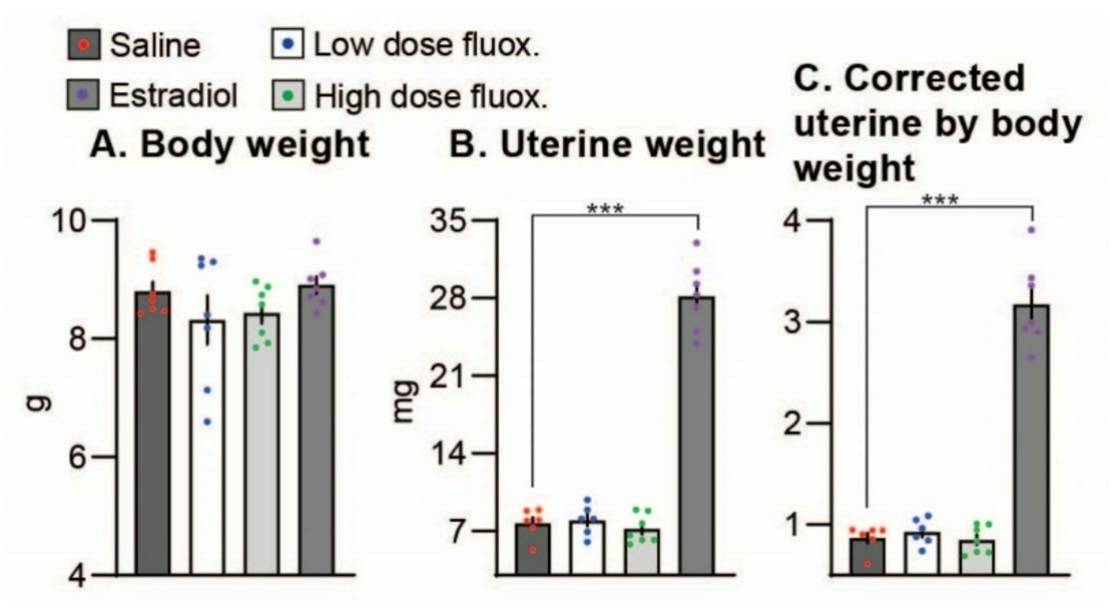
Uterotrophic assay in WT prepubertal (sexually immature) mice. Effect of fluoxetine treatment on (**A**) body weight (**B**) uterine weight (**C**) uterine corrected to body weight. Immature nineteen-day old wild-type (WT) female mice were treated with saline, 17b-estradiol (positive control, 10 µg/kg), and fluoxetine (low [2 mg/kg/d] and high dose [20 mg/kg/d]) for three days and euthanized 24 h after the last treatment (mice were 22-days old at the time of euthanasia). Saline *n* = 7, low dose fluoxetine *n* = 7, high dose fluoxetine *n* = 7, estradiol *n* = 7. ***indicates P value < 0.0001.

The rodent uterotropic response bioassay model is the gold-standard for determining estrogenic activity in vivo, according to the Organization for Economic Cooperation and Development Test Guideline 440^28–30^. The bioassay evaluates the ability of a compound to elicit estrogenic biological activity resulting in increased uterine weight. We performed a uterotrophic assay in sexually immature (prepubertal) female mice to assess the estrogenic activity of fluoxetine. Immature nineteen-day old wild-type (WT) female mice were treated with saline, 17b-estradiol (positive control, 10 µg/kg), and fluoxetine (low and high dose) for three days and euthanized 24 h after the last treatment (mice were 22-days old at the time of euthanasia). Uterine weight and uterine weight corrected by body weight increased in the 17b-estradiol-treated group (*P* < 0.0001) 3.7-fold and 3.9-fold, respectively (Fig. 1). Similarly, 17b-estradiol increased (*P* < 0.0001) uterine thickness (2.3-fold), lumen diameter (5.6-fold), and epithelium height (1.8-fold) compared to the control (not shown). Fluoxetine at either dosage did not affect uterine dynamics (uterine weight, uterine weight corrected by body weight, uterine thickness, lumen diameter, and epithelium height) indicating that fluoxetine does not directly elicit estrogenic activity.

### Experiment 2

**Fig. 2:**
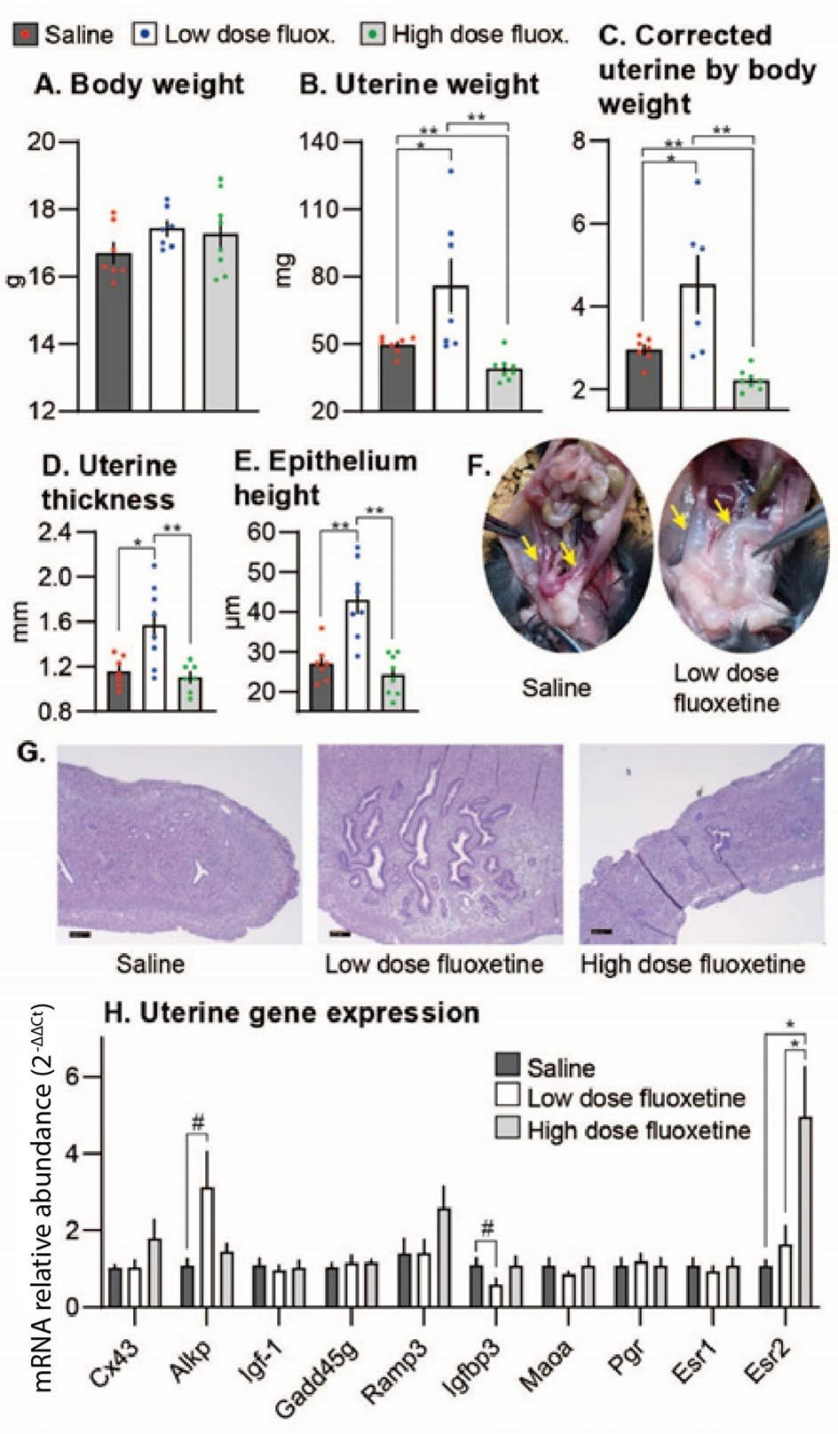
Effect of fluoxetine treatment on WT mice. Sexually mature (6-7-weeks old), virgin WT females were treated with saline or fluoxetine (low [2 mg/kg/d] and high [20 mg/kg/d] dose) from experimental day 0 to 6 and euthanized six hours after the last treatment. (**A**) Body weight (**B**) Uterine weight (**C**) Uterine weight corrected to body weight (**D**) Uterine thickness (**E**) Epithelium height (**F**) Representative images of uterus collected from mice treated with saline and low dose fluoxetine. Arrows indicate uterine horns. (**G**) Representative histological images of the uterus collected from mice treated with saline, low dose fluoxetine, and high dose fluoxetine. Bar = 200 μm. (**H**) Relative gene expression in uterus from mice treated with saline, low dose fluoxetine, and high dose fluoxetine. Saline *n* = 7, low dose fluoxetine *n* = 8, high dose fluoxetine *n* = 8. *indicate *P* < 0.05, **indicates P value < 0.01, #indicates < value < 0.1.

Sexually mature (6-7-weeks old), virgin WT females were treated with saline or fluoxetine (low and high dose) from experimental day 0 to 6 and euthanized six hours after the last treatment. Fluoxetine treatment affected uterine weight. Uterine weight in the low dose fluoxetine group was 53.3% greater compared to control (*P* = 0.04) suggesting endorine-disrupting activity (Fig. 2). In the high dose fluoxetine group uterine weight was 21% lower compared to the control (*P* = 0.005). Uterine weight corrected to body weight followed a similar pattern: greatest in the low dose fluoxetine, intermediate in the control, and lowest in the high dose fluoxetine groups. Expression of alkaline phosphatase (Alkp) was increased 3.1-fold and insulin-like growth factor binding protein 3 (Igfbp3) was downregulated by 47.8% in the low dose group compared to the control. Compared to control and low dose fluoxetine groups, expression of Esr2 in the high dose fluoxetine was increased 4.5- and 3.1-fold, respectively. Expression of other evaluated genes was not different among groups. Uterine morphology in the low fluoxetine group was consistent with modulation of estrogenic activity compared to the control (increased uterine thickness 35.6%, *P* = 0.01; increased epithelial height 58.7%, *P* = 0.001; and increased endometrial grands). Taken together, the low dose fluoxetine treatment elicits estrogenic activity in sexually mature, intact female mice.

### Experiment 3

**Fig. 3:**
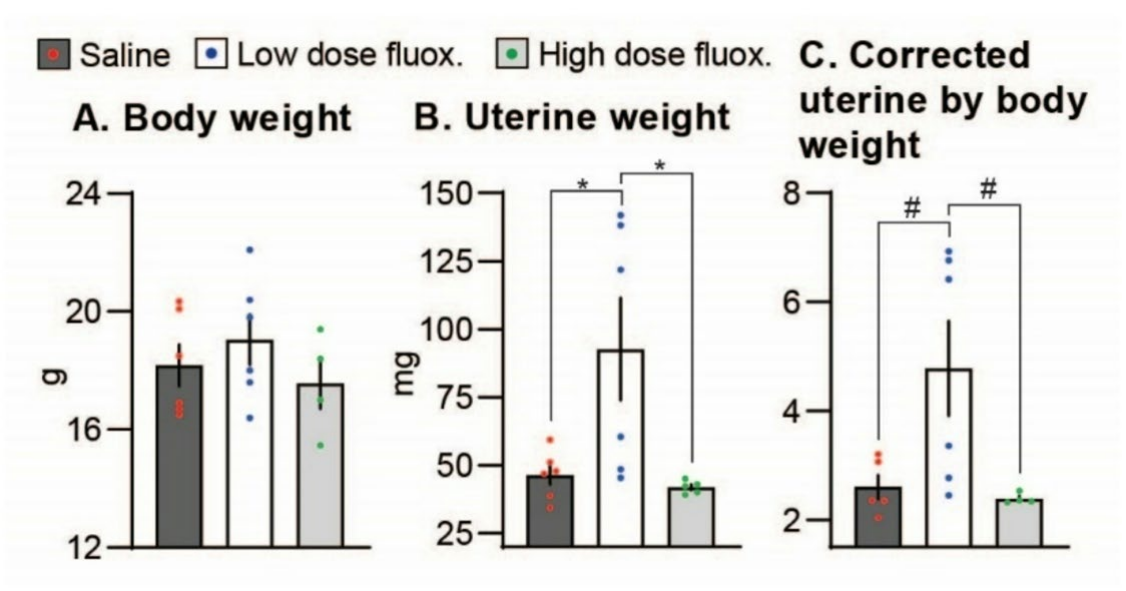
Effect of fluoxetine treatment on (**A**) body weight (**B**) uterine weight (**C**) uterine corrected to body weight in WT pseudopregnant mice. Sexually mature (6-7-weeks old) mice were mated to vasectomized males. After detection of a vaginal plug (experimental day [d] 0), females were treated daily from d0 to d6 with saline or fluoxetine (low [2 mg/kg/d] and high [20 mg/kg/d] dose). Mice were euthanized six hours after the last treatment. Saline *n* = 7, low dose fluoxetine *n* = 6, high dose fluoxetine *n* = 6. *indicates P value < 0.05, #indicates P Value < 0.1.

To investigate whether fluoxetine disrupts endocrine signaling during decidualization, we mated sexually mature (6-weeks old) WT females to vasectomized males. After detection of a vaginal plug (experimental day [d] 0), females were treated daily from d0 to d6 and euthanized six hours after the last treatment. Sample collection on d6 was chosen based on the maximal production of estrogen by the uterus of pregnant/pseudopregnant mice^31^ which coincided with the timeline of observed increased uterine weight in our previous unpublished experiment. In the low dose fluoxetine group, uterine weight was increased 99.7% (*P* = 0.03) and uterine weight corrected to body weight increased 84% (*P* = 0.054) compared to the control group and 120.7% (*P* = 0.03) and 100% (*P* = 0.059), respectively, compared to the high dose fluoxetine group (Fig. 3). Uterine weight and uterine weight corrected by body weight were similar between control and high dose groups. These results are consistent with our findings in experiment 2, suggesting that the low dose of fluoxetine treatment modulates estrogenic activity in pseudopregnant mice.

### Experiment 4

**Fig. 4:**
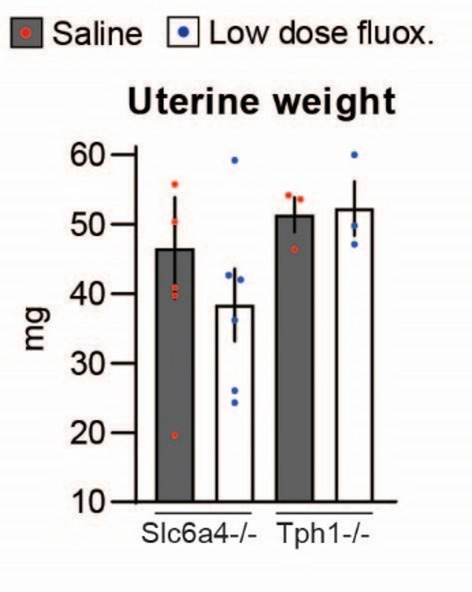
Effect of fluoxetine treatment on uterine weight in serotonin transporter deficient mice (Sert^−/−^) and tryptophan hydroxylase 1 deficient mice (TPH1^−/−^). Sexxually mature mice were treated daily with saline or low dose fluoxetine (2 mg/kg/d) from experimental day 0 to 6 and euthanized six hours after the last treatment. Sert^−/−^ saline *n* = 6, Sert^−/−^ low dose fluoxetine *n* = 6, TPH1^−/−^ saline *n* = 3, TPH1^−/−^ low dose fluoxetine *n* = 3.

To assess whether the uterotropic effects of low dose fluoxetine are dependent on SERT (encoded by Slc6a4), the binding target for fluoxetine, we treated sexually mature (7–8 weeks old), virgin Slc6a4^−/−^ females with saline and low dose fluoxetine. Further, to assess whether the effects of fluoxetine are dependent on peripheral serotonin, we treated sexually mature (7–8 weeks old), virgin Tph1^−/−^ females with saline and low dose fluoxetine. Tryptophan hydroxylase (Tph) is the rate-limiting enzyme for serotonin synthesis; Tph2 is mainly expressed in neuronal tissue, whereas Tph1 is mainly expressed in the periphery^32,33^. Mice deficient in Tph1 are unable to produce peripheral serotonin while brain serotonin synthesis is maintained^32,34–36^. Because only the low dose fluoxetine treatment appeared to have uterotropic actions (Experiments 2 and 3), it was the only dose of fluoxetine used in the mutant mice experiments. Mice were treated daily from experimental day 0 to 6 and euthanized six hours after the last treatment. Body weight was not different among WT, Slc6a4^−/−^, and Tph1^−/−^ mice (*P* > 0.5). Uterine weight and uterine weight corrected by body weight were not affected by fluoxetine treatment in Slc6a4^−/−^ (*P* > 0.3) and Tph1^−/−^ (*P* > 0.8) genetic models (Fig. 4). Similarly, uterine weight corrected by body weight was not affected by fluoxetine treatment in either Slc6a4^−/−^ (*P* > 0.2; saline: 2.8 ± 0.4, fluoxetine: 2.2 ± 0.3) and Tph1^−/−^ (*P* > 0.3; saline: 2.6 ± 0.2, fluoxetine: 3.0 ± 0.2) genetic models.

To further assess the role of SERT function on uterotropic action of fluoxetine, we used Slc6a4^+/−^ mice. The Slc6a4^+/−^ mice have decreased Sert expression but similar SERT function compared to WT counterparts^37^. Therefore, similarly to WT mice, low dose fluoxetine treatment increased uterine weight (61%, *P* = 0.029) and uterine weight corrected by body weight (40%, *P* = 0.054) in Slc6a4^+/−^ (not shown). Taken together, the results in the mutant mouse models suggest the uterotropic actions of fluoxetine are dependent on intact SERT function and circulating serotonin.

## Discussion

Given the widespread use of SSRI, understanding their side effects, particularly those related to reproductive function, is critical for the assessment of their impact on women’s health. The present study demonstrates a role for SSRI on modulation of uterine dynamics. Specifically, a low dose fluoxetine treatment elicited uterotropic response consistent with those of estrogen agonists only in sexually mature, intact WT mice. Although these endocrine disrupting effects of SSRI are side-effects of its intended action as an antidepressant, they are mediated by interaction with its biological target site, SERT, rather than an off-target mechanism. Furthermore, these effects are dependent on SSRI modulation of peripheral, but not neuronal, serotonin signaling. Lastly, the lack of positive uterotropic response of fluoxetine in prepubertal WT mice suggest fluoxetine does not directly bind estrogen receptors to elicit uterotropic activity but modulate signaling of endogenous estrogen. Considered together, although the notion that SSRI elicit estrogenic activity is not novel, we demonstrated for the first time that fluoxetine’s in vivo uterotropic effects are mediated via inhibition of SERT and dependent on endogenous synthesis of estrogen and serotonin.

Uterotrophic assays in immature, prepubertal rodents are used for in vivo assessment of the ability of a substance to elicit biological activity consistent with agonist of estrogen receptor^28,29,38–40^. As the hypothalamic-pituitary-ovarian axis is not functional in prepubertal animals, endogenous estrogen synthesis is minimal. Therefore, if a given substance binds to estrogen receptors, it will stimulate a biological response in target tissues such as the uterus with activation of molecular pathways resulting in increased cell division and growth with a consequent increase in uterine weight^38,39,41^. The lack of uterotropic response in sexually immature mice treated with fluoxetine in the present study differs from the study of Muller and colleagues^17^ when using a similar experimental approach and doses of fluoxetine but in Wistar rats. In both studies, the increase in uterine weight in the estradiol-treated positive control group was about 400% compared to the negative control group. In our study, fluoxetine did not uterotropic response at either tested dose. In contrast, doses of 1.7 and 17 mg/day, but not 0.4 mg/kg, of fluoxetine increased uterine weight by about 50% compared to the control group in Wistar rats^17^. This conflicting result may be related to a differential effect of fluoxetine in mice vs. rats. However, for uterotrophic assays in immature rodents, it is critical that animals have not entered puberty. In our study, all mice used for the uterotrophic assay were born in our colony and were 19-days old at onset of treatment and 22-days old at euthanasia/assessment of uterus weight. This is in contrast to the later onset of treatment and assessment of uterine weight in the Wistar rat study which may have obscured interpretation of the effects of the drug due to interefrance of endogenous estrogen^17^. Nevertheless, it appears that fluoxetine does not bind estrogen receptor to elicit its uterotropic (estrogenic) response.

The increase in uterus weight in sexually mature WT mice at different reproductive stages (nonpregnant cyclic and pseudopregnancy) demonstrates the endocrine disruption effects of fluoxetine modulating uterine dynamics (estrogenic response)^31,42^. The increase in Alkp, a regulator/marker of uterine stromal differentiation, and decreased Igfbp3 support increased estrogen signaling in addition to the histological morphological alteration in the low dose fluoxetine group^31,41^. Furthermore, expression of Igfbp3 has been reported to be inversely related to uterine weight as in the present study^41^. Taken together, the uterotropic response of the low dose fluoxetine treatment is dependent on endogenous synthesis of estrogen. Indeed, fluoxetine treatment has been shown to inhibit serotonin uptake into murine primary granulosa cells^16^ and oocytes^43^ and to increase systemic estradiol in rodents^44^. An important novelty of our study is that the fluoxetine-induced increased estrogen synthesis/signaling is mediated by inhibition of SERT and is serotonin-dependent. This was evident by the lack of uterotropic response in mice lacking serotonin transporter (Slc6a4^−/−^) and mice with depleted peripheral synthesis of serotonin (Tph1^−/−^). The neuronal synthesis of serotonin is likely unaltered as Tph2 is the main tryptophan dehydrogenase in the brain whereas Tph1 is mostly expressed in the periphery^32^. This is consistent with our experiment 1, which further supports the idea that fluoxetine does not direct bind estrogen receptors to elicit its uterotropic (estrogenic) actions. Therefore, the endocrine disrupting effects of SSRI are indirect, that is, dependent on inhibition of SERT and dependent on circulating estrogen and serotonin.

Previous studies have shown that serotonin binding to its receptor 2 A subtype stimulates estrogen synthesis by enhancing aromatase activity, the enzyme responsible for aromatization of androgens into estrogens^22,45–47^. Further studies suggested that other serotonin receptor subtypes may be involved in this mechanism as multiple serotonin receptors are present in granulosa cells and oocyte^16,48^. Serotonin stimulates steroid synthesis and secretion by human and rodent ovarian granulosa cells^47–50^. The SSRI increase circulating serotonin and extends the serotonin availability in the extracellular space by blocking SERT. Our study in mutant mice were critical to demonstrate that the uterotropic effects of SSRI are dependent on the functional expression of SERT and on availability of peripheral serotonin. Therefore, it is possible that the SSRI-induced increase in serotonin availability leads to increased aromatase activity and estrogen synthesis resulting in the increased uterine weight observed in the present study. The effects of SSRI on estrogen synthesis have also been investigated in human adrenocortical carcinoma cell line (H295R) that synthesize all steroid hormones^19,20,51^. Hansen and colleagues^19^ demonstrated that the six commercially available SSRI (fluoxetine, paroxetine, citalopram, escitalopram, sertraline, and fluvoxamine; vilazodone was not approved for use at the time of the study) stimulate estrogen synthesis by enhancing aromatase activity in H295R cells. Similarly, apparent increases in aromatase activity by SSRI have been reported by others using H295R cells^20,51^. Additionally, fluoxetine also appears to stimulate aromatase activity in human trophoblast-like BeWo cells^21,22^. Noteworthy, adrenocortical neoplastic tissues, such as the H295R cell line used in these in vitro experiments, have increased serotonin synthesis phenotype^52^. Therefore, it is likely that SSRI inhibition of SERT in H295R cells increased serotonin signaling which ultimately increased estrogen synthesis in this cell line.

Findings from the present study add to our previous study^18^ in which we demonstrated that treatment with fluoxetine disrupts reproductive function. However, the effects of fluoxetine on endocrine regulation and reproductive function appear to be differently regulated in rodents according to species (mice vs. rats), genetic background, dose, and exposure period^17,18,25,26,53^. For instance, fluoxetine treatment promotes different sexual behavior outcomes in Sprague-Dawley vs. Fishers rats^26^. In the present study, only the low dose of fluoxetine elicited uterotropic response after six days of treatment. However, based on our previous study^18^, treatment with a low dose of fluoxetine does not affect estrous cyclicity and does not affect uterine weight after 14 days of treatment suggesting that the effects of fluoxetine on uterine dynamics may be transient. On the contrary, the high dose of fluoxetine caused interruption of the estrous cycle within days after onset of treatment, along with decreased estrogen signaling by day 14 of treatment as observed by decreased uterine weight and altered uterine gene expression. In the present study, the high dose fluoxetine treatment also decreased uterine weight (experiment 2) suggesting that this dose of fluoxetine causes more abrupt effects on reproductive function with longer-lasting effects. These differences in behavioral response and regulation of reproductive function may be associated, at least in part, to differential modulation of the central nervous system response to the drug, including pharmacokinetics, serotonin transporter binding affinity and distribution, and interactions between serotonin and estrogen systems.

A weakness of the present study is that we were unable to measure circulating concentrations of estrogen due to technical issues with hormonal assays and the available volume of serum collected from mice. Demonstration of increased circulating estrogen along with reproductive cycles and ovarian/uterine function in subjects treated with fluoxetine would provide more robust insight into the endocrine disruption actions of SSRI. Lastly, the effects of SSRI exposure prenatally or during the prepubertal period on later reproductive function post-puberty were not assessed. Santos and colleagues^54^ showed delayed puberty onset (vaginal opening and first estrus) in female rodents exposed to SSRI in utero and during lactation. In contrast, the age of puberty was not affected by fluoxetine but irregular estrous cycles and altered ovarian follicle population were observed in Wistar rats exposed to fluoxetine perinatally^55^. A comprehensive assessment of SSRI exposure at different stages of life and reproductive cycle is warranted.

In conclusion, we demonstrated the endocrine disrupting effects of fluoxetine treatment on sexually mature WT mice as indicated by the uterotropic response. This effect was SERT-mediated and serotonin-dependent indicating that SSRI act through SERT to elicit its uterotropic responses. Importantly, the uterotropic response of fluoxetine occurred only at the low-dose and is dependent on endogenous synthesis of estrogen. These findings are important given the increasing number of women at reproductive age that are exposed to SSRI medication and the potential side effects associated with SSRI use, including sexual dysfunction.

## Materials and methods

### Animals

All experimental procedures were approved by the Research Animal Care and Use Committee at the University of Wisconsin-Madison performed under protocol number A005789-A01 and in accordance with ARRIVE guidelines. Wild-type C57BL/6J (strain 000664) and Slc6a4^−/−^ (strain 008355, C57BL/6 background) were obtained from Jackson Laboratories (Bar Harbor, ME) and colonies were maintained in our facility. We have also maintained a colony of Tph1^−/−^ mice^34^. Mice were individually housed in a controlled environmental facility for biological research in the Animal and Dairy Sciences Department vivarium at the University of Wisconsin-Madison. The vivarium was maintained at a temperature of 25 °C and a humidity of 50% to 60%, with a 12:12 h light-dark cycle with *ad libitum* water and food (LabDiet 5015, TestDiet, Richmond, IN).

### Fluoxetine treatments

In each experiment, mice were randomly allocated to a treatment group. Low dose fluoxetine (2 mg/kg/d) and high dose fluoxetine (20 mg/kg/d) were administered via intraperitoneal injection. Fluoxetine hydrochloride (F312; Sigma-Aldrich, St. Louis, MO) was dissolved in saline. The control group received intraperitoneal saline alone. We have previously used these doses of fluoxetine in mice^56^. The low dose (2 mg/kg/d) treatment results in fluoxetine concentrations similar to that of humans undergoing fluoxetine treatment for diverse psychological conditions^56^. The high dose (20 mg/kg/d) results in greater systemic fluoxetine concentrations than typically observed in humans although it is routinely used in mice studies^57,58^. Using the body surface area calculations for translation of mouse doses to human doses, a 60 kg human, with a 1.6 m^2^ surface area, 37 *km* factor and a conversion factor of 1, taking a dose ranging between 10 and 90 mg of fluoxetine per day would be a fluoxetine dose in the range of 2.05–18.45 mg/kg/d in a mouse^59,60^. This indicates that the fluoxetine doses used in the present experiments encompass a range of fluoxetine doses in humans highlighting the clinical relevance of our studies.

The duration of treatment for the uterotropic assay in immature (pre-pubertal) mice was based on international standards for screening potential endocrine disrupting compounds. For sexually mature mice, the duration of treatment was based on our previous observation of endocrine disrupting effects of fluoxetine^18^.

### Blood and tissue collection

Mice were euthanized approximately 6 h after the last treatment with carbon dioxide followed by cervical dislocation. Cardiac blood was collected immediately after euthanasia, and the uterus was excised and weighed. One uterine horn along with the ovaries were fixed in 4% paraformaldehyde overnight and stored in 70% ethanol until histological processing. Histology samples were embedded in paraffin, sectioned into 8 μm sections, stained with conventional hematoxylin-eosin and observed by light microscopy for image collection and analyzed qualitatively by a single technician unaware of treatment group. Images were obtained with a Zeiss AX10 microscope and images captured with a Basler acA2440-35ucMED camera. Uterine thickness and epithelium height were measured (ImageJ) in areas of the epithelium where the nucleus and basement membrane of single epithelial cells were seen^30,61^. Four measurements were made in each uterine section; at least three sections were evaluated for each mouse. The other uterine horn was snap frozen in liquid nitrogen and stored at -80 °C and used for evaluation of gene expression.

### Extraction of RNA, complementary DNA, and quantitative PCR

Extraction of RNA was performed with Quick-RNA MiniPrep Plus kit (Zymo Research, CA, USA) as described by the manufacturer and quantified by spectrometry with a NanoDrop 2000 spectrophotometer (Thermo Scientific, USA). Complementary DNA (cDNA) was synthesized using the High-Capacity cDNA Reverse Transcription Kit (Applied Biosystems, CA, USA) as described by the manufacturer using 1 µg of total RNA. The cDNA was used directly for quantitative real-time PCR (qRT-PCR). The qRT-PCR reactions were carried out on an CFX Connect Real-Time PCR system (Bio-Rad Life Science, CA, USA) using a master mix that contained a total volume of 10.5 µL per tube consisting of 6.25 µL of SsoFast EvaGreen Supermix (Bio-Rad Laboratories Inc., CA, USA), 3.25 µL of nuclease-free water, and 0.5 µL of forward and reverse primers (10 µM). Two µL of cDNA at a 1:5 dilution was added to the master mix for a total reaction volume of 12.5 µL. All samples were evaluated in duplicate. The reactions were initiated with preincubation at 95 °C for 3 min followed by 42 cycles of denaturation (95 °C for 10 s) and annealing and extension (60 °C for 30 s).

Targeted genes were selected to assess the transcription of estrogen-responsive genes^18,31,41^. The primer sequences for targeted genes were synthesized by Integrated DNA Technologies Inc. (CA, USA) and have been previously reported by our laboratory^18^. Efficiencies of qRT-PCR for amplification of targeted genes were determined in our laboratory and ranged from 95% to 107%. The amplification data obtained from the qRT-PCR were the cycle threshold (Ct) for each mRNA and these was used to calculate the mRNA relative abundance of each sample by the 2^−∆∆Ct^ method^62^ using the saline group as baseline and the geometric mean of the housekeeping genes 36b4 and Hrpt1.

### Statistical analysis

All statistical analyses were performed with SAS (version 9.4; SAS Institute Inc., Cary, North Carolina, USA). Data were analyzed with the PROC MIXED procedure using one-way ANOVA, each treatment group was compared to the control group. Residuals with deviations from assumptions of normality and/or homogeneity of variance were transformed into square root, logarithms, or ranks. A probability of ≤ 0.05 indicated a difference was significant and a probability between > 0.05 and ≤ 0.1 indicated tendency significance. Data are presented as the mean ± standard error of mean (SEM).

## Data Availability

The data underlying this article are available in the article.
